# Capnometry in suspected pulmonary embolism with positive D-dimer in the field

**DOI:** 10.1186/cc8197

**Published:** 2009-12-08

**Authors:** Tadeja Hernja Rumpf, Miljenko Križmarić, Štefek Grmec

**Affiliations:** 1 University Clinical Centre Maribor, Ljubljanska 5, 2000 Maribor, Slovenia; 2 Faculty of Health Sciences, University of Maribor, Žitna ulica 15, 2000 Maribor, Slovenia; 3 Centre for Emergency Medicine Maribor, Ulica talcev 9, 2000 Maribor, Slovenia; 4 Faculty of Medicine, University of Ljubljana, Vrazov trg 2, 1000 Ljubljana, Slovenia; 5 Faculty of Medicine, University of Maribor, Slomškov trg 15, 2000 Maribor, Slovenia

## Abstract

**Introduction:**

Pulmonary embolism (PE) is one of the greatest diagnostic challenges in prehospital emergency setting. Most patients with suspected PE have a positive D-dimer and undergo diagnostic testing. Excluding PE with additional non-invasive tests would reduce the need for further imaging tests. We aimed to determine the effectiveness of combination of clinical probability and end-tidal carbon dioxide (PetCO_2_) for evaluation of suspected PE with abnormal concentrations of D-dimer in prehospital emergency setting.

**Methods:**

We assessed clinical probability of PE and PetCO_2 _measurement in 100 consecutive patients with suspected PE and positive D-dimer in the field. PetCO_2 _> 28 mmHg was considered as the best cut-off point. PE was excluded or confirmed by hospital physicians in the University Clinical Center Maribor by computer tomography (CT), ventilation/perfusion scan echocardiography and pulmonary angiography.

**Results:**

PE was confirmed in 41 patients. PetCO_2 _had a sensitivity of 92.6% (95% CI, 79 to 98%), a negative predictive value of 94.2% (95% CI, 83 to 99%), a specificity of 83% (95% CI, 71 to 91%) and a positive predictive value of 79.2% (95% CI, 65 to 89%). Thirty-five patients (35%) had both a low (PE unlikely) clinical probability and a normal PetCO_2 _(sensitivity: 100%, 95% CI: 89 to 100%) and twenty-eight patients (28%) had both a high clinical probability (PE likely) and abnormal PetCO_2 _(specificity: 93.2%, 95% CI: 83 to 98%).

**Conclusions:**

The combination of clinical probability and PetCO_2_ may safely rule out PE in patients with suspected PE and positive D-dimer in the prehospital setting.

## Introduction

Pulmonary embolism (PE) is a common disorder with substantial associated morbidity and mortality [1,2]. It typically has a nonspecific clinical presentation and often poses a significant diagnostic challenge [3,4]. Accurate diagnosis in the prehospital emergency setting is critical because 30-day mortality in patients in whom the diagnosis is initially missed is 17% [5]. Several non-invasive tests have been introduced to reduce the need for further diagnostic tests in patients with suspected PE. The D-dimer test is usually performed first because it can safely rule out PE and thus, reduce the need for further testing [6]. However, because of its poor specificity, especially in elderly patients, patients with cancer, hospitalized patients and pregnant women, the D-dimer test excludes PE in only 30% of patients [7-11]. The first step in safely using the D-dimer test is to determine the patient's risk of PE. The most frequently used clinical prediction rule is the Canadian rule, developed by Wells and colleagues as shown in Table 1[6,7]. This rule has been validated extensively using both a three-category (low, moderate or high clinical probability) and a two-category scheme (PE likely or unlikely) [12,13]. Alternative non-invasive tests that can be used in the prehospital setting are required. Capnometry and capnography are reliable diagnostic and prognostic tools for a variety of conditions [14-16]. PE significantly decreases alveolar carbon dioxide (CO_2_) content [17-19]. It obstructs blood flow to a normally ventilated area of lung, producing a locally high ventilation, low perfusion relation, therefore increasing alveolar dead space [20]. Gas exhaled from this unperfussed lung unit contains little CO_2 _and therefore reduces the partial pressure of end-tidal carbon dioxide (PetCO_2_) of the whole lung in relation to the partial pressure of arterial CO_2 _(PaCO_2_). Alveolar dead space fraction ((arterial CO_2 _- end-tidal CO_2_)/arterial CO_2_) has insufficient sensitivity to exclude PE safely [21-23]. Some previous studies demonstrated the use of a combination of alveolar dead space fraction measurement and D-dimer testing, and this combination has been suggested to be superior to either tool used in isolation [21-23]. Sanchez and colleagues demonstrated the use of a combination of alveolar dead space fraction and clinical probability [24]. In recently published guidelines on the diagnosis and management of acute PE, authors concluded that negative D-dimer safely excluded PE in patients with low clinical probability ('PE unlikely' patients). The negative predictive value of D-dimer was high. In patients with high clinical probability ('PE likely' patients) normal results did not safely exclude PE. The positive predictive value of D-dimer was low, so D-dimer was not useful for confirming PE [25].

**Table 1:** Clinical probability (the Wells score) of pulmonary embolism

Wells score*	
**Variable**	**Points**
Previous DVT or PE	+ 1.5
Recent surgery or immobilization	+ 1.5
Cancer	+ 1
Haemoptysis	+ 1
Heart rate > 100 beats/min	+ 1.5
Clinical signs of DVT	+ 3
Alternative diagnosis less likely than PE	+ 3
**Clinical probability (3 levels)**	**Total**
Low	0-1
Intermediate	2-6
High	> _7
**Clinical probability (2 levels)**	**Total**
PE unlikely	0-4
PE likely	> 4

*Wells score [7].

DVT = deep vein thrombosis; PE = pulmonary embolism.

Therefore, we hypothesized that the combination of PetCO_2 _and clinical probability with positive D-dimer test could improve diagnostic accuracy in the prehospital setting in patients with suspected PE. We wanted to determine if the combination of two level clinical probability assessment and PetCO_2_ measurement could confirm or exclude PE in patients with an abnormal D-dimer test.

The aim of this study is to determine whether PetCO_2_ improves sensitivity for exclusion of PE in unlikely patients with abnormal D-dimer results, and confirms PE with high specificity in PE likely patients in the prehospital setting.

## Materials and methods

### Setting

Between October 2004 and December 2008, this prospective cohort observational study was performed in the prehospital emergency setting (Center for Emergency Medicine Maribor, Slovenia, Europe). The study was approved by the Ethical Review Board of the Ministry of Health of Slovenia. We did not obtain patient consent as a part of the protocol. We argued successfully to the Ethical Review Board that the protocol posed minimal risk to patients and the board deemed consent not to be required. The study was conducted in the city of Maribor and adjacent rural areas encompassing a population of 200,000 inhabitants spread over an area of 780 km^2^. The emergency medical service system is accessed through a single emergency number (112). The system includes two prehospital emergency teams with advanced life support (ALS) capability, two basic life support (BLS) teams, and during the daytime - from April to October - a rescuer on a motorcycle. Each ALS team is comprised of one emergency physician and two additional personnel who are either registered nurses, medical technicians, or a combination of the two; all with training in advanced cardiac life support. Each BLS team is comprised of two nurses or registered nurses and the motorcycle rescuer who is a nurse or a registered nurse, all with BLS training and able to provide electrical defibrillation, chest compressions, ventilation, and oxygenation before arrival of the ALS team. If the call refers to a life-threatening emergency, the ALS team is concomitantly dispatched. On occasion, the ALS team is called by the BLS team after on site recognition of a life-threatening emergency.

### Patients

All consecutive patients presented with clinically suspected PE and a positive D-dimer test were eligible for inclusion in the study (n = 170).

Inclusion criteria were: age older than 18 years; a clinical suspicion of acute PE, defined as acute chest pain, new onset or worsening dyspnea without other obvious causes, and/or a collapse with the symptoms of obstructive shock; and a positive D-dimer test assessed by the rapid quantitative test - CARDIAC D-Dimer measurements (≥ 500 mg/L).

Exclusion criteria were: inability to participate; ongoing anticoagulation for other diseases (e.g. atrial fibrillation); patients under intubation; history of renal insufficiency; and/or in the final stages of a terminal illness.

After prehospital care, all patients were admitted to the University Clinical Center Maribor and followed until discharge.

### Design of the study

This prospective cohort observational study was performed in the prehospital emergency setting (Center for Emergency Medicine Maribor, Slovenia, Europe) between October 2004 and December 2008.

Clinical probability of PE (PE likely or unlikely) was assessed using a prediction rule by Wells and colleagues (Table 1) and was followed by plasma D-dimer test (Cardiac D-dimer measurements - Roche Diagnostics, Mannheim, Germany). D-dimer was measured in all patients with initial clinical suspicion of acute PE, both in the PE likely and PE unlikely groups. The components of the score by Wells and colleagues were collected by prehospital emergency physicians and recorded in a protocol. During initial evaluation (before application of medicine), a 5 mL sample of venous blood was collected into a tube containing calcium disodium edetate for the measurement of D-dimer. The level of D-dimer was measured with a portable automatic device (Cardiac Reader, Roche Diagnostics, Mannheim, Germany), and recorded in the paper collection form (protocol). The patients with normal D-dimer concentration (< 0.5 μg/ml) were not included in the study. We analysed and followed up only the patients with abnormal D-dimer results. Arterial blood gas analysis and other laboratory tests were performed in the hospital laboratory. PetCO_2 _measurements were carried out in all patients with abnormal D-dimer concentrations. PetCO_2 _was obtained by quantitative capnometry, performed with a Lifepak 12 (Medtronic Physiocontrol, Corporate Headquarters, Redmond, WA, USA); PetCO_2 _value (an average value of the first three measurements in the first minute after nasal measurement) was registered. The final hospital diagnosis of PE (at the University Clinical Center Maribor) was confirmed by hospital physicians blinded to the values of PetCO_2 _and prehospital D-dimer results, using the reference standard definition for PE in accordance with instruments, including computed tomography (CT), ventilation/perfusion scan, echocardiography and pulmonary angiography. Prehospital emergency physicians and physicians at admission to hospital (emergency department of internal medicine) were not blinded to the results of D-dimer and PetCO_2 _because these are the routine tests in our prehospital emergency care. In addition, the investigators did not collaborate in making the final diagnosis.

Pulmonary embolism evaluation was considered positive by satisfying one of the following conditions: 1) positive CT, 2) high probability V/Q lung scan or 3) positive pulmonary angiography.

### Statistical analysis

Univariate comparison was made with chi-squared test for categorical variables and Student's t-test for continuous variables. Univariate analysis was performed for all variables pertinent to diagnose PE, and multivariate analysis was performed to identify potential predictor variables of a final diagnosis of PE (variables from univariate analysis with a *P *value less than 0.05 for entry into model). The area under the receiver operating characteristic curve (AUROC) was used for diagnostic accuracy of quantitative capnometry in confirming PE in patients with positive D-dimer in the prehospital emergency setting.

To evaluate the diagnostic performances of the PetCO_2 _testing, sensitivity, specificity, positive and negative predictive values and their 95% confidence intervals (CI) were calculated according to standard methods for proportions. Calculation was performed for the whole group of patients tested, then according to high (PE likely) or low (PE unlikely) clinical probability. Sensitivity was defined as the number of patients with a positive result on PetCO_2 _divided by the number of patients with PE. Specificity was defined as the number of patients with a negative result of PetCO_2 _divided by the number of patients without PE. All analyses were performed using SPSS 15.0 for Windows (SPSS Institute, Chicago, IL, USA).

## Results

**Figure 1:**
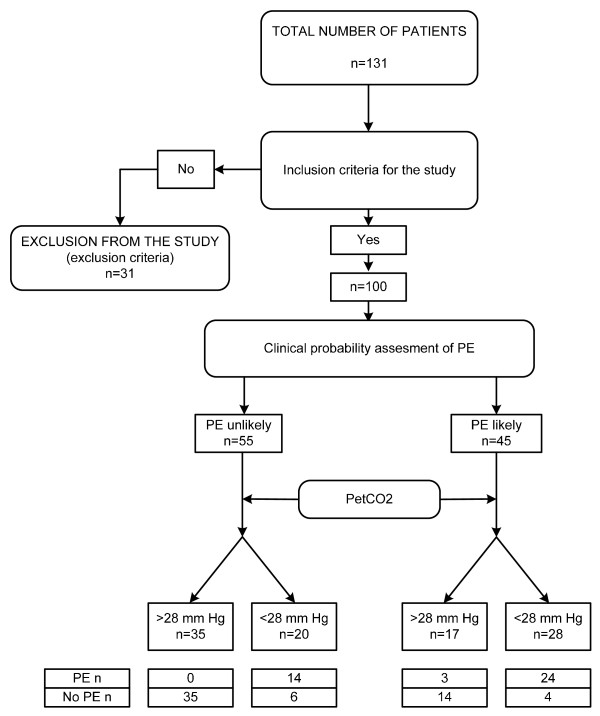
The flow diagram of recruitment, exclusion and subsequent grouping of all patients in the study. PE = pulmonary embolism; PetCO_2 _= partial pressure of end-tidal carbon dioxide.

Between October 2004 and December 2008, 131 patients with suspected PE and a positive D-dimer test were enrolled. Thirty-one patients were excluded because of anticoagulant treatment for more than 48 hours before inclusion (n = 10), inability to participate (n = 12) and receiving mechanical ventilation (n = 9). Recruitment, exclusion and subsequent grouping of all patients are shown in Figure 1. The baseline clinical and demographic variables of the study populations are displayed in Table 2. For the identification of the final diagnosis of PE, we examined 37 variables (Table 2) and 11 variables remained statistically significant after analysis. Variables from univariate analysis (with *P *< 0.05) were included into a model of multivariable analysis with logistic regression for identification of potential predictor variables of a final diagnosis of PE. Finally, after multivariable logistic regression analysis six variables were defined as independent predictor variables: PaCO_2_, partial pressure of arterial oxygen (PaO_2_), D-dimer, PetCO_2_, cyanosis, previous deep vein thrombosis and/or PE (Table 3).

**Table 2:** Univariate analysis for all demographic and clinical variables pertinent to diagnosis of pulmonary embolism (n = 100)

Variable**	PE (n = 41)	No PE (n = 59)	*P *value#
**Demographic data**			
Age (mean ± SD)	71 ± 13	70 ± 11	0.752
Gender (M/F)	15/26	33/26	0.057
Previous DVT or PE (Y/N)	10/31	2/57	**0.001**
Smoker (Y/N)	5/36	25/34	**0.001**
**Thromboembolic risk factors**			
Surgery or fracture within 1 month (Y/N)	9/32	6/53	0.105
Malignancy (Y/N)	10/31	6/53	0.056
Hormone therapy (Y/N)	2/39	1/58	0.359
Palpitation (Y/N)	8/33	18/41	0.218
Calf pain (Y/N)	9/32	10/49	0.531
Relatively asymptomatic (Y/N)	2/39	0/59	0.087
Thrombophlebitis (Y/N)	2/39	0/59	0.087
Unilateral leg swelling (Y/N)	6/35	2/57	0.041
Cyanosis (Y/N)	10/31	1/58	**< 0.001**
Chronic venous insufficiency (Y/N)	1/40	2/57	0.784
COPD (Y/N)	10/31	10/49	0.360
Heart failure (Y/N)	18/23	38/21	**0.042**
Hemiparesis (Y/N)	2/39	4/55	0.694
Immobilization (Y/N)	4/37	2/57	0.187
Suspected DVT	6/35	3/56	0.101
Family history of venous thromboembolism (Y/N)	2/39	0/59	0.087
**Clinical symptoms and signs**			
Syncope (Y/N)	19/22	28/31	0.912
Pulse (1/min)	104 ± 18	93 ± 13	**0.001**
PaCO_2 _(mmHg)	36 ± 4	41 ± 6	**< 0.001**
PaO_2 _(mmHg)	9 ± 1	12 ± 1	**< 0.001**
Dyspnea (sudden onset) (Y/N)	39/2	57/2	0.709
Pleural chest pain (Y/N)	27/14	48/11	0.078
Hemoptysis (Y/N)	2/39	2/57	0.709
Sweating (Y/N)	4/37	3/56	0.368
SpO_2 _(%)	87 ± 6	88 ± 7	0.655
D-Dimer (mg/L)	2010 ± 804	1238 ± 692	**< 0.001**
Cough (Y/N)	12/29	18/41	0.894
PetCO_2 _(mmHg)	25 ± 2	32 ± 4	**< 0.001**
Body temperature (°C)	36.8 ± 0.4	36.9 ± 0.4	0.641
Systolic BP (mmHg)	113 ± 21	122 ± 30	**0.116**
Diastolic BP (mmHg)	71 ± 12	71 ± 16	**0.794**
Crackles on auscultation (Y/N)	18/23	25/34	**0.879**
Respiratory rate (1/min)	23 ± 5	21 ± 2	**0.024**

BP = blood pressure; COPD = chronic obstructive pulmonary disease; DVT = deep venous thrombosis; F = female; M = male; N = no; PaCO_2 _= partial pressure of arterial carbon dioxide; PaO_2 _= partial pressure of arterial oxygen; PE = pulmonary embolism; PetCO_2 _= partial pressure of end-tidal carbon dioxide; SD = standard deviation; SpO_2 _= peripheral oxygen saturation; Y = yes;

** Results are presented as mean +/- standard deviation for normally distributed data or ratio or percentage of other variables.

**# **Univariate comparison was made with chi-square test for categorical variables and t test for continuous variables. For evaluation of diagnostic accuracy, patients were divided into two groups: with PE and without PE.

**Table 3:** Logistic regression analysis of factors used for confirmation of PE in patients with positive D-dimer in prehospital emergency setting

Factor	OR (95% CI)**	*P *value#
PaCO_2_	9.8 (4.2-15.1)	< 0.001
PaO_2_	14.1(6.9-27.4)	< 0.001
D-dimer	15.3 (6.3-25.8)	< 0.001
PetCO_2_	7.4 (2.8-17.8)	< 0.001
Cianosis	6.2 (1.8-13.1)	0.013
Previous DVT or/and PE	6.8 (1.5-11.7)	0.021
Smoker	0.14 (0.04-0.34)	< 0.001

CI = confidence interval; DVT = deep venous thrombosis; PaCO_2 _= partial pressure of arterial carbon dioxide; PaO_2 _= partial pressure of arterial oxygen; PE = pulmonary embolism; PetCO_2 _= partial pressure of end-tidal carbon dioxide; OR = odds ratio.

** Univariable screening was performed on clinical, historical and biochemical variables to identify potential predictors of PE. Odds ratios for the presence of PE were generated and expressed with 95% CI.

# Multivariable analysis with logistic regression was used to identify potential predictor variables of a final diagnosis of PE (variables from univariate analysis with *P *< 0.05. For entry into model).

PE was diagnosed during the initial diagnostic work up by a positive spiral CT in 78 patients, a high probability ventilation/perfusion scan in 20 patients and pulmonary angiography in 2 patients. These patients were considered to have PE for the analysis of the diagnostic accuracy of PetCO_2_. Thus, PE was confirmed in 41 patients (41%) and excluded in the remaining 59 patients (59%) on the basis of the results of initial diagnostic work up. Among 31 patients who met exclusion criteria for PE, no one had confirmed diagnosis of PE. Five of 41 patients with PE died, because of recurrent PE during hospitalization. Two of 59 patients without PE died: 1 of septic shock and 1 of cardiac arrest.

### End-tidal carbon dioxide

**Figure 2:**
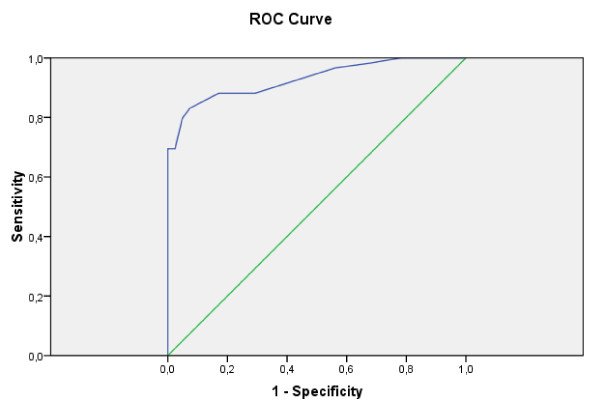
Receiver-operator characteristics (ROC) curve for end-tidal carbon dioxide.

Receiver operating characteristics analysis selected 28 mmHg as the optimal cut-off for PetCO_2 _(Figure 2). The AUROC curve for PetCO_2 _is 0.929 (95% CI = 0.881 to 0.977).

Thirty-eight of the 41 patients (92.6%) with PE had abnormal PetCO_2 _of less than 28 mmHg compared with 10 of the 59 patients (16.9%) without PE. A PetCO_2 _above 28 mmHg excluded PE with a sensitivity of 92.6% (95% CI = 79 to 98%), a negative predictive value of 94.2% (95% CI = 83 to 99%), a specificity of 83% (95% CI = 71 to 91%) and a positive predictive value of 79.2% (95% CI = 65 to 89%).

Thirty-five patients had a low (PE unlikely) clinical probability and PetCO_2 _above 28 mmHg. This combination excluded PE with a sensitivity of 100% (95% CI = 89 to 100%), a negative predictive value of 100% (95% CI = 88 to 100%). Twenty-eight patients had a high clinical probability (PE likely) and a PetCO_2 _below 28 mmHg. This combination had a specificity of 93.2% (95% CI = 83 to 98%) and a sensitivity of 58.5% (95% CI = 42 to 73%) for PE (Table 4).

**Table 4:** Combination of clinical probability and PetCO_2_ measurement in patients with positive D-dimer and suspected pulmonary embolism

Combinations	PE	No PE
PE likely OR PetCO_2_ < 28 mmHg (suggests PE possible)	41	24
PE unlikely AND PetCO_2_ > 28 mmHg (Rule out PE)	0	35

PE likely AND PetCO_2_ < 28 mmHg (suggests PE possible)	24	4
PE unlikely OR PetCO_2_ > 28 mmHg (Rule out PE)	17	55

PE = pulmonary embolism; PetCO_2 _= partial pressure of end-tidal carbon dioxide.

## Discussion

In our study, we have demonstrated that the combination of PetCO_2 _of more than 28 mmHg and low clinical probability (PE unlikely) is a potentially safe method for excluding PE in patients with suspected PE and positive D-dimer test in the prehospital setting. The results also suggest that the measurement of PetCO_2 _alone has a lower negative predictive value (94%; 95% CI = 83 to 99%) than the previously mentioned combination of tests (100%; 95% CI = 89 to 100%).

In our study we found that the combination of high clinical probability (PE likely) and a PetCO_2 _of less than 28 mmHg had 93.2% specificity (95% CI = 83 to 98%) for the confirmation of PE.

Some studies [21-23] have evaluated the diagnostic accuracy of capnography in patients with suspected PE. The multicenter study by Kline and colleagues [21] calculated sensitivity as 67.2% (95% CI = 55.0 to 77.5%) and specificity as 76.3% (95% CI = 71.2 to 85.6%). Rodger and colleagues [22] calculated sensitivity as 79.5% (95% CI = 63.5 to 90.7%) and a specificity of 70.3% (95% CI = 61.2 to 78%). A negative predictive value varied from 90.7% [22] to 91.9% [21]. Hogg and colleagues [23] calculated sensitivity as 100% (95% CI = 84.5 to 100%) but a low specificity of 22.7% (95% CI = 18.8 to 27.2%). The combination of a normal alveolar dead space fraction and normal D-dimer concentration excluded PE with a sensitivity ranging from 90.5% to 98.4% [21-23]. Sanchez and colleagues [24] combined alveolar dead space fraction and clinical probability assessment in patients with a positive D-dimer. The combination of a normal alveolar dead space fraction and a low clinical probability excluded PE with a sensitivity of 99.1% (95% CI = 94.9 to 100%) and a negative predictive value of 97.8% (95% CI = 88.2 to 99.9%). Our study shows similar results as this study, the difference being that we combined PetCO_2 _and clinical probability assessment in patient with a positive D-dimer.

Our study suggests that a simple method of nasal measurement of PetCO_2 _in combination with clinical evaluation can safely exclude PE without blood gas analysis and calculations of PaCO_2 _- PetCO_2 _gradient (unpractical for diagnostics in the field).

What impact do these results have on patient care, and what patient benefit is derived from the out-of-hospital study? The primary goal of our observational, prospective study was to find out the diagnostic rule of PetCO_2 _in patients with suspected PE and abnormal D-dimer results. The study showed that these results could be useful in the emergency department. Corwin and colleagues [26] and Hirai and colleagues [27] reported that emergency physicians did not use D-dimer effectively to determine the need for CT or angiography in the evaluation of acute PE. The use of quantitative D-dimer in combination with PetCO_2 _in the prehospital setting would decrease unnecessary imaging and irradiation, costs and time for patients seen in the admission department. The results from the field can help in diagnostic decisions. The prehospital emergency physician can organize direct transport from the field to pulmonary vascular imaging. Squizzato and colleagues [28] showed in a meta-analysis that 928 patients with symptomatic PE were treated completely as outpatients or discharged early.

In previous recommendations for the diagnosis of PE [25], bedside echocardiography was recommended for high-risk PE (presence of shock or persistent hypotension). Mansencal and colleagues [29] found that echocardiography (using a portable ultrasound device) is a reliable method for screening patients with suspected PE, especially in patients with dyspnea or with high clinical probability. The prehospital point-of-care ultrasound is reality [30], and its findings can help in thrombolytic therapy in the field. Thrombolytic therapy is the first-line treatment in patients with high-risk PE presenting with cardiogenic shock and/or persistent arterial hypotension because it rapidly exerts beneficial effects on hemodynamic parameters.

We used the prediction rule described by Wells and colleagues. There is no clear difference in diagnostic performance between using another clinical prediction rules, but the score by Wells and colleagues has been more extensively validated.

However, this study has some limitations. Firstly, prehospital emergency physicians and hospital physicans at admission were not blinded to the values of PetCO_2 _and the D-dimer test as assessed by the rapid quantitative test because capnometry represents the routine procedure in our prehospital management.

Secondly, the study was realized in a single emergency center with a relatively small sample size. Thirdly, all patients would have had the reference standard (CT, pulmonary angiography or a normal ventilation/perfusion scan result). Finally, severe additional factors may have an impact of the reliability of PetCO_2 _(some patient hyperventilated, had periodic breathing or had ventilation/perfusion mismatch).

## Conclusions

We conclude that implementing D-dimer and quantitative capnometry into standard daily prehospital care of acute dyspnea exerted a beneficial effort on the PE diagnosis and the eventually need for immediately treatment. However, the diagnostic accuracy of these methods should be confirmed in a larger multicenter study in the field (in combination with the point-of-care prehospital ultrasound) for determination of whether these findings can be safely incorporated.

In conclusion according to the results of our study, PetCO_2 _measurement has been demonstrated as a useful adjunct to standard clinical evaluation and identification of PE in patients with positive D-dimer tests in the prehospital setting. The combination of clinical assessment and PetCO_2 _measurement in patients with low clinical probability (PE unlikely) has better diagnostic value than D-dimer in combination with clinical assessment. The combination of PetCO_2 _concentrations and high clinical probability (PE likely) is a potentially safe method for confirmation of PE in patients with suspected PE and positive D-dimer test in prehospital setting.

## Key messages

• The combination of PetCO_2 _concentration higher than 28 mmHg and low clinical probability (PE unlikely) is a potentially safe method for excluding PE in patients with suspected PE and positive D-dimer test in the prehospital setting.

• The measurement of PetCO_2 _alone has a lower negative predictive value than the combination of tests.

• The combination of PetCO_2 _concentrations less than 28 mmHg and high clinical probability (PE likely) is a potentially safe method for confirmation of PE in patients with suspected PE and positive D-dimer test in prehospital setting.

## Abbreviations

ALS: advanced life support; AUROC: area under the receiver operating characteristic curve; BLS: basic life support; CI: confidence interval; CO_2_: carbon dioxide; CT: computed tomography; PaCO_2_: partial pressure of arterial carbon dioxide; PaO_2_: partial pressure of arterial oxygen; PE: pulmonary embolism; PetCO_2_: partial pressure of end-tidal carbon dioxide.

## Competing interests

The authors declare that they have no competing interests.

## Authors' contributions

THR designed the study, collected the data and wrote the draft of the manuscript. MK made the statistical analysis and wrote the draft of the manuscript. ŠG designed the study, made the statistical analysis and wrote the draft of the manuscript. All authors finalized and approved the manuscript.
